# Monoclonal regulatory T cells provide insights into T cell suppression

**DOI:** 10.1038/srep25758

**Published:** 2016-05-23

**Authors:** Céline Gubser, Mathias Schmaler, Simona W. Rossi, Ed Palmer

**Affiliations:** 1 Departments of Biomedicine and Nephrology, University Hospital Basel and University of Basel, 4031 Basel, Switzerland; 2 Laboratory of Experimental Immunology, Department of Biomedicine, University Hospital Basel and University of Basel, 4031 Basel, Switzerland; 3 Laboratory of Regulatory Immunology Department of Biomedicine, University Hospital Basel and University of Basel, 4031 Basel, Switzerland

## Abstract

Regulatory T cells (Tregs) have a crucial role in maintaining lymphocyte homeostasis. However an understanding of how Tregs function at a cellular and molecular level has not yet been fully elucidated. Here, we make use of a T cell receptor (TCR) transgenic, Rag^−/−^ mouse expressing a Forkhead-Box-Protein P3 (Foxp3) transgene. This mouse provides a source of monoclonal CD4^+^ Foxp3^+^ T cells with a defined specificity. Here we show that monoclonal B3K506 Tregs are functional *in vitro* and *in vivo* and clearly require cognate antigen to be suppressive. We further show that the strength of Treg stimulation determines the strength of Treg mediated suppression. Finally we analysed various suppressive mechanisms used by monoclonal Tregs and found that Treg-Tconv proximity is a parameter, which correlates with enhanced suppression.

CD4^+^ CD25^+^ Foxp3^+^ regulatory T cells (Tregs) are critically involved in the maintenance of lymphocyte homeostasis^1^. Absence of functional Tregs leads to massive cytokine secretion and multi-organ lymphocytic infiltration resulting in polyendocrinopathies and enteropathies (i.e. colitis), a condition termed “scurfy” in the mouse^2^ and immunodysregulation polyendocrinopathy enteropathy X-linked syndrome (IPEX) in the human^3^.

The Treg TCR repertoire has been described as enriched in self-reactive TCRs, specific for tissue-restricted antigens presented in the periphery^4^,^5^,^6^. Encounter of peripheral self-antigens allows constant activation and survival of Tregs^7^,^8^. Despite the self-reactivity of Tregs, the role of TCR signalling in Treg biology has been controversial and is still not fully understood. Numerous reports support the idea that TCR signalling in Tregs is uncoupled from the signalling pathways described in conventional T cells^9^,^10^. The idea that TCR stimulation is blunted or deviated to maintain an anergic, suppressive Treg phenotype has received experimental support. These results raised questions whether TCR signalling is even required for Treg mediated suppression^11^. However recent data, using a model where the TCR can be deleted in peripheral Tregs, show that continuous expression and signalling through the TCR is required for effective suppression to occur *in vivo*^12^. Loss of TCR in peripheral Tregs resulted in lethal autoimmunity^12^.

Previous work characterized Tregs and described their suppressive mechanisms. These mechanisms can be divided into two main groups, i.e. those that depend on cell-cell contact and those that do not. Studies using transwell suppression assays, suggested a predominant role for cell-cell contact dependent suppressive mechanisms^13^,^14^. However, a failure to observe suppression in these experiments could be explained by the inability of diffusible Treg-derived suppressive molecules to function in the relatively large volume of the *in vitro* culture. Theoretically, suppression might depend on Treg-secreted molecules but additionally require proximity between Tregs and Tconv cells^15^. Cytotoxic T-lymphocyte-associated Protein 4 (CTLA-4) is constitutively expressed on Tregs^16^ and thought to be important for suppression. Mice with Treg specific CTLA-4 deficiency suffer from spontaneous development of systemic lymphoproliferation and fatal T cell autoimmunity^17^. It has been suggested that Tregs initiate the catabolism of tryptophan in dendritic cells through a CD80/86-CTLA-4 dependent mechanism, generating metabolites, which convert naïve CD4 Tconvs into induced Tregs (iTregs) with tolerogenic properties^18^,^19^,^20^. It was shown that CTLA-4 down regulates co-stimulatory molecules CD80 and CD86 on antigen presenting cells (APCs) via trans-endocytosis^21^,^22^,^23^. By diminishing the APC’s capacity to costimulate T cells, Tregs may prevent priming of Tconvs^24^,^25^. Another suppressive mechanism involves high expression of lymphocyte function-associated antigen 1 (LFA-1) on Tregs, which has been proposed to augment the physical interaction between Tregs and APCs. In this way, Tregs may out compete Tconvs for “space” on the APC^26^.

Considering mechanisms that are cell-contact independent, Tregs secrete TGFβ and IL-10 immunosuppressive cytokines, which have been shown to control Tconv proliferation^27^,^28^. Treg derived TGFβ was shown to convert naïve T cell precursors into iTregs^29^. However, the role of TGFβ in Treg suppression remains controversial since Tregs mediate suppression of Tconvs from TGFβRII^−/−^ and Smad3^−/−^ mice^30^. In addition, Tregs from neonatal TGFβ^−/−^ mice retained their suppressive capacity^30^. Gut Tregs were shown to secrete IL-10, which was required for mucosal immune homeostasis and control of colitis^31^,^32^,^33^. However, Treg specific IL-10 deficient mice do not suffer from systemic autoimmunity per se; rather they fail to control immune responses at mucosal/environmental interfaces (i.e. gut, lung)^34^. Furthermore, blocking either IL-10 or TGFβ failed to abrogate Treg mediated suppression *in vitro*^35^.

Tregs have additionally been shown to disrupt Tconv metabolism through cyclic adenosine monophosphate (cAMP)^36^,^37^ or by scavenging cytokines^38^. In this regard, Tregs constitutively express high levels of the IL-2 receptor alpha chain (CD25), which might be able to scavenge IL-2 from Tconvs, preventing their full activation^39^. On the other hand, two observations question the idea that Tregs function by consuming (i.e. scavenging) Tconv-generated IL-2: (i) Tregs from CD25^−/−^ mice are suppressive *in vitro*^39^ and (ii) mice with a deletion of the IL-2Rβ (CD122) gene limited to peripheral Tregs don’t suffer from autoimmune disorders^40^. However more recent work mitigated these interpretations. CD122 deficient Tregs still respond to IL-2, albeit to a diminished extent^41^. CD25 deficient Tconvs exhibit a compensatory up-regulation of CD122 and the common gamma chain (CD132)^42^, which renders them capable of responding to, and consuming IL-2. This may occur on CD25 deficient Tregs as well^15^. Finally, another line of evidence for IL-2 consumption as a suppressive mechanism lies in the fact that addition of exogenous IL-2 abrogates suppression (*in vitro*)^13^,^14^.

Here, we attempt to re-assess some of the mechanisms of Treg mediated suppression and the TCR’s contribution. To investigate the mechanism of suppression it would be valuable to have a source of monoclonal Tregs. A difficulty of most monoclonal TCR Tg mice is, they contain very few Tregs. To circumvent this problem we bred Foxp3 transgenic mice^43^ to animals carrying the MHC II restricted B3K506 TCR transgene^44^. Using these B3K506 TCR Tg Rag^−/−^ Foxp3 Tg mice as a source of monoclonal Tregs (B3K506 Tregs), we analysed several parameters for their influence on Treg mediated suppression. The results indicate that the strength of Treg stimulation by peptide-MHC (pMHC) antigen determines the strength of Treg mediated suppression. Furthermore the data show that Treg-Tconv proximity could be an important component of suppression.

## Results

### Generating mice producing monoclonal Tregs

**Figure 1:**
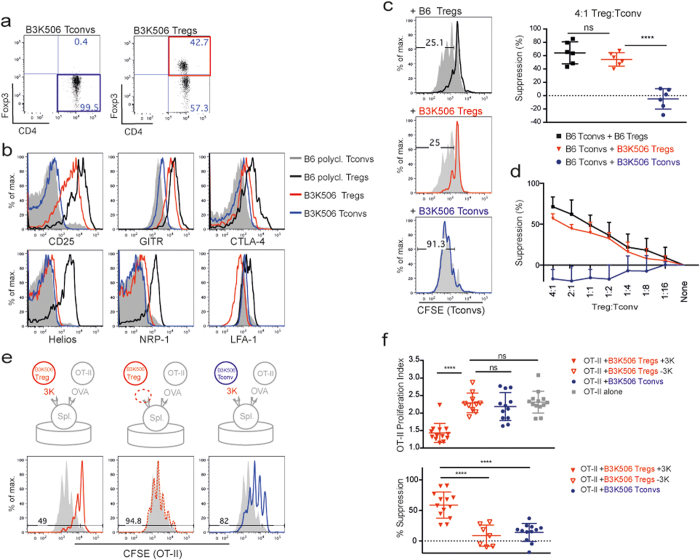
(**a**) Representative flow cytometry plots of CD4^+^ LN T cells from B3K506TCR Tg Rag^−/−^ (B3K506 Tconv) and B3K506 TCR Tg Foxp3 Tg Rag^−/−^ (B3K506 Treg) mice are shown. Numbers in plots depict % of cells in the quadrant. (**b**) Representative histograms show surface marker expression on monoclonal LN B3K506 Tregs (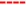), polyclonal B6 Foxp3^EGFP^ Tregs (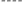), polyclonal B6 Tconvs (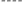) and monoclonal LN B3K506 Tconvs (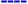), n = 4. (**c**) *In vitro* anti-CD3ε suppression assays. Representative proliferation of polyclonal B6 CD4^+^ Tconvs co-cultured with polyclonal B6 Foxp3^EGFP^ Tregs (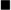), monoclonal B3K506 Tregs (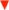) or B3K506 Tconv (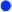) at a 1_Tconv_/4_Treg_ ratio. Grey histograms indicate proliferation of B6 CD4^+^ Tconvs cultured without Tregs. Numbers in histograms depict % proliferated cells. Graph shows mean % suppression + /− SD at 72h, n = 5. (**d**) *In vitro* anti-CD3ε suppression assays. Capacity of monoclonal B3K506 Tregs (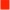), polyclonal B6 Foxp3^EGFP^ Tregs (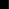) and monoclonal B3K506 Tconv (negative control; 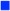) to suppress proliferation of polyclonal B6 CD4^+^ T cells at varying Treg/Tconv ratios. Data show mean % suppression + /− SD, n = 3. **(e/f)** Antigen stimulated suppression assays. (**e**) CFSE labelled OT-II Tconvs were co-cultured with B3K506 Tregs at a 1_Tconv_/4_Treg_ ratio in the presence (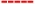) or absence (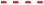) of 3K peptide, or with B3K506 Tconv (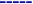) in the presence of 3K peptide. All cultures contained OVA peptide. OT-II Tconv proliferation in the absence of Tregs is shown (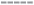). Numbers in histograms represent % proliferated cells. (**f**) OT-II Tconv proliferation index and % suppression in co-cultures described in E). Monoclonal B3K506 Tregs + 3K peptide (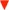); monoclonal B3K506 Tregs −3K peptide (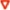); monoclonal B3K506 Tconvs (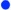); OT-II Tconvs alone (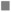). All cultures contained OVA peptide. Data show mean proliferation index + /− SD, n = 9–12. Statistical significance was calculated using a one-way ANOVA and subsequently Bonferroni’s multiple comparisons test. NS = p > 0.05, *p ≤ 0.05, **p ≤ 0.01, ***p ≤ 0.001, ****p ≤ 0.0001.

A Foxp3 transgene^43^ was backcrossed to TCR transgenic B3K506 Rag^−/−^ mice^44^ to generate Rag^−/−^, B3K506 TCR transgenic, Foxp3 transgenic (B3K506 Treg) mice. Forty-50% of T cells in this strain were Foxp3^+^ (Fig. 1a). This contrasts with T cells from Rag^−/−^, B3K506 TCR transgenic (B3K506 Tconv) mice, where >99% T cells were Foxp3-negative (Tconvs) (Fig. 1a). Compared to poly-clonal B6 Tregs, monoclonal B3K506 Tregs expressed slightly reduced levels of CD25, glucocorticoid-induced TNFR-related protein (GITR) and LFA-1, and clearly decreased amounts of CTLA-4 (Fig. 1b). B3K506 Tregs neither expressed Helios nor neuropilin 1 (NRP-1) (Fig. 1b).

B3K506 Treg mice were lymphopenic and were therefore treated with IL-2 bound to JES6-1 monoclonal antibodies for specific enrichment of Tregs (see Supplementary Fig. S1). The capacity of monoclonal B3K506 Tregs to suppress polyclonal CD4^+^ conventional T cells (Tconvs) was assessed *in vitro* using anti-CD3 as a stimulus (Fig. 1c). No significant difference between the suppressive capacity of monoclonal B3K506 and polyclonal B6 Tregs was observed. As expected, B3K506 Tconv cells were not suppressive (Fig. 1c,d). We also established a peptide specific suppression assay using monoclonal B3K506 Tregs and monoclonal CD4^+^ OT-II Tconvs (the 3K peptide is specific for B3K506 Tregs and OVA_323-339_ peptide is specific for OT-II Tconvs: Fig. 1e,f). B3K506 Tregs clearly required TCR stimulation to induce suppressive activity, while antigen stimulated B3K506 Tconvs do not induce suppression (Fig. 1e,f).

### B3K506 monoclonal Tregs require antigen recognition to mediate suppression

**Figure 2:**
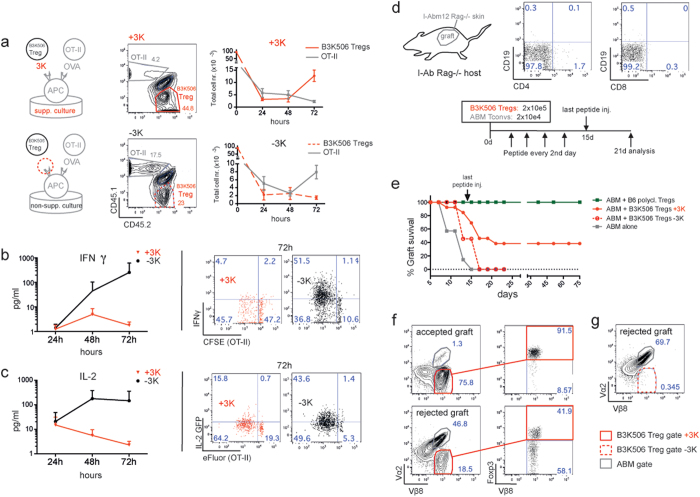
(**a**) Stimulated B3K506 Tregs outcompete OT-II Tconvs, *in vitro*. Representative flow cytometry plots show % of OT-II Tconvs and B3K506 Tregs cocultured at a 1_Tconv_/4_Treg_ ratio in suppressive (with 3K peptide) and non-suppressive (without 3K peptide) co-cultures at 72h. Numbers in the plots represent % of live cells in gate. Graphs show mean numbers + /− SD of live OT-II Tconvs (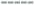) and B3K506 Tregs (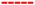) from co-cultures described in A), n = 3. (**b**) Left: Graph depicts mean IFNγ (pg/ml) + /− SD in supernatants of suppressive (red) and non-suppressive (black) co-cultures at a 1_Tconv_/4_Treg_ ratio, n = 3. Right: Representative flow cytometry plots show intracellular IFNγ staining of OT-II Tconvs in suppressive (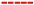) vs. non-suppressive (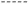) co-cultures. (**c**) Left: Graph depicts mean IL-2 (pg/ml) + /− SD in supernatants of suppressive (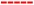) and non-suppressive (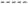) co-cultures at a 1_Tconv_/4_Treg_ ratio, n = 3. Right: Representative flow cytometry plots show GFP expression from eFluor labelled OT-II IL-2^GFP^ reporter Tconvs in suppressive (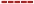) vs. non-suppressive (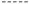) co-cultures. (**d**) Experimental design of *in vivo* suppression assay. Dot plots show PBMCs from a B6 I-A^b^ Rag^−/−^ host (recipient mouse) which had been grafted with skin from a B6 I-A^bm12^ Rag^−/−^ donor mouse prior to T-cell transfer. (**e**) Skin from B6 I-A^bm12^ Rag^−/−^ donor mouse was transplanted onto B6 I-A^b^ Rag^−/−^ mice and allowed to heal for 14 days. The following day mice were injected with 2 × 10e4 ABM (I-A^bm12^ specific) Tconvs along with polyclonal B6 Foxp3^EGFP^ Tregs (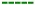) or monoclonal B3K506 Tregs (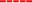 and 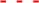). An additional group (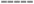) was injected with ABM Tconvs alone. One group (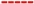) received 3K peptide (30 μg per mouse, i.p. every second day) until day 15. Graph shows % graft survival + /− SD vs. time. Each group contains 5 to 8 mice and results are pooled from 2 independent experiments. (**f**) Representative flow cytometry plots show ABM Tconvs and B3K506 Tregs from the draining LN of skin-grafted mice. Cells in upper plots are from a mouse which received 3K peptide and accepted the skin graft, while cells in lower plots are from a mouse which rejected it’s graft, despite receiving the Treg cognate peptide, 3K. Number in the plots depict % of cells in each gate/quadrant. (**g**) Representative flow cytometry plot showing ABM Tconvs and B3K506 Tregs from the draining LN of skin-grafted mice, which did not receive 3K peptide treatment and rejected their graft. Numbers in the plot depict % of cells in each gate/quadrant.

We wondered whether antigen stimulated Tregs influence the survival/expansion and functional profile of co-cultured OT-II Tconvs. Therefore, we analysed live cell numbers of B3K506 Tregs and OT-II Tconvs as well as cytokine concentrations in supernatants of suppressive (+3K peptide) and non-suppressive (−3K peptide) cultures (Fig. 2a–c). In suppressive cultures, OT-II Tconvs proliferate poorly (Fig. 2a, top) and secrete reduced amounts of IFNγ and IL-2 (Fig. 2b,c). This is consistent with the observation that suppressed OT-II Tconvs are less activated and express less CD25 and CD69 than non-suppressed OT-II Tconvs (see Supplementary Fig. S1). Furthermore, B3K506 Tregs accumulated in antigen-stimulated cultures but not in cultures lacking 3K peptide (Fig. 2a).

B3K506 Tregs also required antigen stimulation for suppressive activity *in vivo*. We made use of a previously established tolerance model^45^ where skin grafts from B6 I-A^bm12^ Rag^−/−^ donor mice were transplanted onto B6 I-A^b^ Rag^−/−^ mice and 14d later challenged with a combination of adoptively transferred ABM (I-A^bm12^ specific) TCR Tg Tconvs and B3K506 Tregs in the presence or absence of the cognate 3K peptide (Fig. 2d,e). Administering 3K peptide until day 15 induced graft survival lasting ≥75 days in 50% of the transplanted mice. All transplanted animals, which didn’t receive 3K peptide rejected their grafts by day 17. A few animals from both groups (receiving or not receiving 3K peptide) where sacrificed and analysed on day 21 post adoptive T cell transfer. Mice tolerating their graft (Fig. 2f, top row) contained many B3K506 Tregs and only few ABM Tconvs in the draining LN. Mice, which rejected their graft in spite of receiving 3K peptide contained many ABM Tconvs but fewer Foxp3^+^ B3K506 Tregs (Fig. 2f, bottom row). Finally mice, which did not receive 3K peptide rejected their grafts and contained almost no B3K506 Tregs (Fig. 2g). These results show that antigen recognition by the Treg is clearly required for suppression, both *in vitro* and *in vivo*.

### Suppression correlates with strength of antigen stimulation, Treg numbers, and CD25 expression

**Figure 3:**
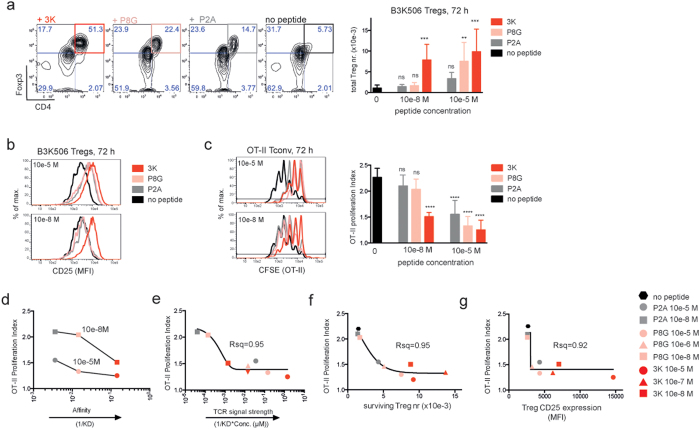
(**a**) Representative flow cytometry plots show B3K506 Tregs from suppressive co-cultures stimulated with various B3K506 altered peptide ligands at a 1_Tconv_/4_Treg_ ratio at 72h. Numbers in the plots depict % of cells in each quadrant. Bar graph shows mean number of B3K506 Tregs + /−SD from these cultures at 72 h, n = 5. (**b**) Representative histograms show CD25 expression (MFI) on CD4^high^ Foxp3^high^ B3K506 Tregs from suppressive co-cultures stimulated with various altered peptide ligands or no peptide at a 1_Tconv_/4_Treg_ ratio at 72 h. (**c**) Representative histograms depict proliferation of CFSE labelled OT-II Tconvs form suppressive co-cultures described in A). Bar graph shows mean proliferation index + /−SD of OT-II Tconvs at 72 h, n = 4–8. **(a–c)** Statistical significance was calculated using a one-way ANOVA and subsequently a Bonferroni’s multiple comparisons test. NS = p > 0.05, *p ≤ 0.05, **p ≤ 0.01, ***p ≤ 0.001, ****p ≤ 0.0001 (**d**) Curves show correlation between pMHC affinity (1/KD) for the B3K506 TCR and OT-II proliferation index from suppressive co-cultures described in A). APCs were pulsed with 10e-8M peptides (◽) or 10e-5M peptides (⚬). (**e**) Curve shows correlation between OT-II Tconv proliferation index and B3K506 Treg stimulus strength, which combines pMHC affinity and peptide concentration and is defined as [1/KD x peptide conc. (μM)] from suppressive co-cultures described in A). Curve is a nonlinear fit with variable slope (4 parameters). (**f**) Correlation between OT-II Tconv proliferation index and number of surviving Tregs from suppressive co-cultures described in A). Curve is a nonlinear fit with variable slope (4 parameters). (**g**) Correlation between OT-II Tconv proliferation index and CD25 expression (MFI) on surviving Tregs from suppressive co-cultures described in A). Curve is a nonlinear fit with variable slope (4 parameters).

To further investigate the role of antigen recognition in suppression, B3K506 Tregs were stimulated with several altered peptide ligands. At various times, cultures were assessed for the frequency, absolute cell number (Fig. 3a), CD25 expression (Fig. 3b) and suppressive capacity (Fig. 3C) of CD4^high^, Foxp3^high^ B3K506 Tregs. When used at a concentration of 10e-8M to 10e-5M, stimulation with the high affinity ligand 3K resulted in high numbers of surviving B3K506 Tregs and significant OT-II Tconv suppression (Fig. 3a,c). However, only high concentrations (10e-6 to 10e-5M) of the intermediate affinity ligand, P8G induced Treg survival and significant suppression (Fig. 3a,c). B3K506 Treg survival and suppression were poorly induced by the threshold affinity ligand, P2A (Fig. 3a). However, some suppression of OT-II proliferation could still be observed in co-cultures stimulated with high (10e-5M) P2A concentrations in the cultures (Fig. 3c). All peptide ligands were similarly suppressive when applied at their EC50 concentration as measured by CD69 up regulation (see Supplementary Fig. S2).

The suppressive capacity of B3K506 Tregs clearly correlated with pMHC affinity and peptide concentration (Fig. 3d) as well as with TCR signal strength, a parameter, which combines pMHC affinity for the TCR and peptide concentration (Fig. 3e). Suppression also correlated with the number of surviving B3K506 Tregs (Fig. 3f) and the amount of CD25 they express (Fig. 3g). High Treg cell numbers can compensate for low CD25 expression (e.g.10e-7M 3K, compare red triangles in 3f and 3g) and vice versa (e.g.10e-5M 3K, compare red circles in 3f and 3g). Finally, there is a clear threshold below which, low Treg cell numbers and CD25 expression fail to induce suppression. These results strongly suggest that the strength of Treg stimulation by its cognate antigen drives suppression through induction of Treg expansion, survival and CD25 expression.

### Suppression is enhanced by Treg and Tconv proximity

**Figure 4:**
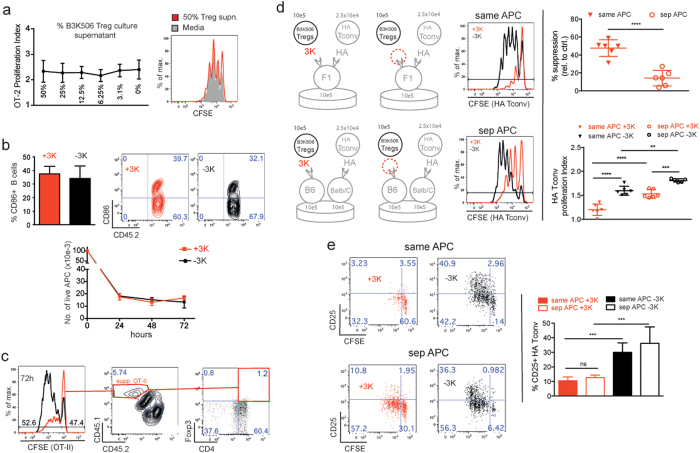
(**a**) Supernatants from stimulated Treg cultures are not suppressive. Graph depicts mean proliferation index + /− SEM of OT-II Tconvs separately cultured in various concentrations of supernatant from separately stimulated B3K506 Treg cultures, n = 3. Representative histograms depict proliferation of OT-II Tconvs cultured in media containing 50% Treg supernatant (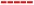) or in media alone (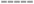). (**b**) Tregs do not affect CD86 expression on APCs or APC cell numbers. Top: Bar graph depicts % of CD86^+^ splenic B cell APCs from suppressive (with 3K peptide, 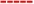) and non-suppressive (without 3K peptide, 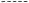) co-cultures. Representative flow cytometry plots show CD86 expression on live, CD19^+^ CD4^−^ splenic B cell APCs from these cultures at 72h. Numbers in the plots depict % of cells in each quadrant. Bottom: Graph shows mean number of live splenic B cell APCs + /− SD vs. time in suppressive vs. non-suppressive co-cultures at various times, n = 3. (**c**) B3K506 Tregs do not convert OT-II Tconvs into induced Tregs (iTregs). Representative flow cytometry plots show Foxp3 and CD4 expression on OT-II Tconvs from suppressive co-cultures at a 1_Tconv_/4_Treg_ ratio at 72 h. Numbers in the plots depict % of cells in each quadrant or gate. (**d**) Suppression is enhanced when Treg and Tconv antigens are presented on the same APC. Left: Representative histograms show proliferation of CFSE labelled HA Tconvs co-cultured with B3K506 Tregs at a 1_Tconv_/4_Treg_ ratio. HA Tconvs and B3K506 Tregs encounter their cognate ligand either on the same (top) or on separate (bottom) splenic B cell APCs (see diagram). Suppressive cultures (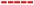) were compared to non-suppressive cultures where 3K peptide was omitted (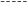). Right: Graph shows mean % suppression (top) and mean proliferation index (bottom) + /−SD of HA-Tconvs co-cultured in the different conditions described in D, n = 4–5. (**e**) Left: Representative flow cytometry plots show CD25 expression on HA Tconvs in cultures described in D). Numbers in the plots represent % of cells in each quadrant. Right: Bar graph depicts mean % CD25^**+**^ HA-Tconvs + /−SD in these cultures at 72 h, n = 4.

We also examined the roles of various suppressive mechanisms using this experimental system. Culture supernatants from separately cultured antigen stimulated B3K506 Tregs, did not suppress the antigen driven expansion of (separately cultured) antigen stimulated OT-IIs, which argues against the presence of soluble suppressive molecules produced at effective concentrations in this system (Fig. 4a). In suppressive (with 3K peptide) and non-suppressive (without 3K peptide) co-cultures, CD86 expression on splenic B cell APCs and surviving splenic B cell numbers were similar (Fig. 4b). This argues that a Treg mediated decrease of CD86 on surviving APCs (i.e. via trans endocytosis) or a Treg mediated killing of APCs was not obviously required for suppression in these cultures. Furthermore, B3K506 Tregs did not convert OT-II Tconvs into iTregs (Fig. 4c) even though B3K506 Tregs expressed high levels of surface latency-associated peptide of TGF-β (LAP) (see Supplementary Fig. S2)

Finally, we wondered whether Tregs and Tconv needed to encounter their cognate peptide on the same APC for effective suppression to occur. To avoid cross-presentation of the Treg and Tconv peptides, we used I-E^d^ restricted HA transgenic Tconvs, whose cognate peptide, HA cannot be presented by I-A^b^. In this system, peptide cross presentation and alloreactions were not evident (see Supplementary Fig. S2). I-A^b^ restricted B3K506 Tregs and I-E^d^ restricted HA Tconvs were co-cultured with B6xBalb/c F1 splenic B cell APCs loaded with both the 3K and HA peptides. This arrangement was referred to as “same APC”. Alternatively, B3K506 Tregs and HA Tconvs were co-cultured with B6 splenic B cell APCs loaded with 3K only and Balb/c splenic B cells loaded with HA peptide only. This arrangement was called “separate APCs”. Results show that suppression was enhanced when both antigens were presented on the same APC (Fig. 4d). However, antigens presented on separate APCs were able to support some degree of suppression, as up regulation of CD25 on HA Tconvs was suppressed in these separate APC co-cultures (Fig. 4e). Taken together, these data suggest that increased proximity between Tregs and Tconvs improves suppression.

## Discussion

Here we show that monoclonal B3K506 Tregs are functional *in vitro* and *in vivo*; furthermore, Tregs clearly require cognate antigen stimulation to be suppressive. Our data also show that the strength of Treg TCR stimulation correlates with the extent of Treg mediated suppression. Increasing the affinity and/or concentration of Treg antigen drives Treg proliferation and CD25 expression, which is likely related to the extent of suppression in this system.

B3K506 Treg mice are lymphopenic and only 40–50% of CD4 T cells are Foxp3^+^ (Fig. 1a). To increase Treg numbers, we injected mice with IL-2 coupled to the monoclonal antibody JES6-1 (Suppl. Fig. 1d,e). Although this particular IL-2/mAb complex was reported to preferentially expand Tregs *in vivo*^46^, this treatment expanded both Foxp3^+^ and Foxp3^−^ B3K506 T cells in B3K506 TCR Tg Rag−/− Foxp3 Tg mice (see Supplementary Fig. S1). Unlike polyclonal, thymus derived Tregs from B6 mice, B3K506 (monoclonal) Tregs expressed neither Helios nor NRP-1. Thymic Tregs in polyclonal mice are thought to be generated through agonist selection^5^,^6^ and may acquire Helios and NRP-1 expression following a high affinity encounter with self-antigen^47^. In contrast, thymocytes bearing the B3K506 TCR are normally positively selected in the B6 genetic background but are directed into the Treg lineage due to expression of the Foxp3 transgene. As B3K506 monoclonal Tregs do not encounter a high affinity self-antigen during their development, they are likely Helios^−^ and NRP1^− ^47,48 (Fig. 1b).

Similar to what was reported for polyclonal Tregs^14^, we observed decreased IFNγ and IL-2 cytokine levels in culture supernatants from suppressive co-cultures containing stimulated B3K506 monoclonal Tregs (Fig. 2b,c). Although suppressed OT-II Tconvs produced very little IFNγ (as measured by intracellular staining), suppression of IL-2 production, as measured by expression of an IL-2-GFP reporter was incomplete (Fig. 2c). In the presence of cognate antigen, B3K506 Tregs expanded between 48 and 72h (Fig. 2a). This contradicts previous work describing a requirement for Treg anergy to achieve effective suppression^13^. Using a previously described skin-graft-transplantation model^45^ we observed that B3K506 monoclonal Tregs are less suppressive than polyclonal Tregs (50% vs. 100% graft survival, respectively) (Fig. 2d–g). This might be explained by a short half-life of 3K peptide *in vivo*, leading to suboptimal Treg activation in the host. However, recipient mice that had accepted their skin graft achieved a stable (75d) tolerance and did not require peptide administration past day 15.

To investigate the role of TCR signal strength in B3K506 Treg suppression, we used three altered peptide ligands (i.e. 3K, P8G, P2A). Although they display an affinity hierarchy for the B3K506 TCR (3K >P8G >P2A) all three pMHC antigens are above the affinity threshold for negative selection^49^ (Fig. 3a–g). All three peptides induced suppression when used at their EC50 concentration for CD69 up-regulation (see Supplementary Fig. S2). However, low affinity peptides were more suppressive when used at increased concentrations. Increased peptide concentration could compensate for decreased affinity to some extent (Fig. 3c). Overall, suppressive capacity correlates with pMHC affinity, peptide concentration, TCR signal strength, B3K506 Treg proliferation and CD25 expression (Fig. 3d–g).

In accordance with published data^50^, we did not detect a soluble suppressive molecule in culture supernatants from antigen stimulated B3K506 Tregs (Fig. 4a). However suppressive cytokines e.g. IL-10, IL-35 and TGFβ or tryptophan metabolites, granzyme B and adenosine may be too diluted or unstable to be suppressive in a relatively large culture volume (200 μl). In this experimental system, CTLA-4 mediated trans-endocytosis of CD86^21^,^22^,^23^ was not observed (Fig. 4b). This mechanism of suppression likely plays a minor role in our system. Suppressive mechanisms involving the induction of APC apoptosis can also be excluded due to the fact that the numbers of surviving APCs in suppressive and non-suppressive co-cultures are similar (Fig. 4b).

Finally, although B3K506 Tregs from suppressive co-cultures expressed surface latency-associated peptide of TGF-β (LAP) (see Supplementary Fig. S2), conversion of OT-II Tconvs into iTregs was not observed (Fig. 4c). This mechanism of suppression is not evident in our experimental system.

Previously reported experiments using transwell culture settings failed to observe suppression^13^,^14^. The authors concluded that Treg-to-Tconv-cell-contact has a predominant role in mediating suppression. Along this line, we designed an experiment where I-A^b^ restricted B3K506 Tregs and I-E^d^ restricted HA Tconvs were co-cultured with B6xBALB/c F_1_ APCs presenting both Treg and Tconv peptides or with separate B6 and BALB/c APCs, each presenting the Treg or the Tconv peptides, respectively (Fig. 4d). In this system, neither peptide cross presentation nor cross-reactivity of each T cell on the inappropriate APC was evident (see Supplementary Fig. S2). Suppression is enhanced when Tregs and Tconvs received antigen stimulation from F1 APCs presenting both peptides. However, partial suppression was detected when cells were cultured on separate APCs. Using separate APCs for each antigen, HA Tconvs were able to proliferate to some degree but did not upregulate CD25 (Fig. 4e).

There is likely a combination of more than one suppressive mechanism, which could explain these results. Suppression may be mediated by a combination of processes, some of which function more efficiently at short distances (e.g. IL-2 scavenging or contact mediated suppression); other mechanisms such as the secretion of suppressive molecules might function over longer distances, i.e. even when the proximity between Tregs and Tconvs is reduced. Nevertheless the data (Fig. 4d) favor the idea that suppression is more efficient when the Treg and Tconv have an increased chance to interact on the same APC, i.e. an APC presenting both the Tconv and the Treg antigens.

In summary, we observed that suppression requires high Treg numbers expressing high amounts of surface CD25 (Fig. 3) and is enhanced by close proximity between Tregs and Tconvs (Fig. 4d,e). These results, along with the observation that addition of exogenous IL-2 abrogates suppression *in vitro*^13^,^14^ (see Supplementary Fig. S2), are consistent with the ability of Tregs to scavenge IL-2 which might account for their capacity to suppress Tconvs. Supporting this idea are the observations that Tregs continuously, and even more so upon TCR and IL-2 signalling, express CD25 (Fig. 3b and see Supplementary Fig. S2). Moreover, due to the direct repression of the IL-2 promoter by Foxp3, Tregs are unable to produce autocrine IL-2^51^. A potential, reliable source of paracrine IL-2 could be a neighbouring CD4^+^ Tconv cell encountering low affinity self-antigen present in the periphery. The uptake of excess paracrine IL-2 by Tregs has beneficial effects on the maintenance of lymphocyte homeostasis: (i) by consuming IL-2, the Treg receives a survival signal and maintains elevated CD25 expression (Suppl. Fig. 2C), and (ii) by decreasing the amount of IL-2 available to Tconvs, Tregs limit the ability of Tconvs to respond to self-antigen.

However, there are a few reports in the literature arguing against IL-2 scavenging as a major mechanism of suppression. Tregs from CD25^−/−^ mice are suppressive *in vitro*^39^ and mice with a specific deletion of CD122 in peripheral Tregs do not suffer from autoimmune disorders^40^. However these arguments have been mitigated by more recent work showing that CD122 deficient Tregs still respond, albeit to a diminished extent, to IL-2 signalling^41^ and Tregs from CD25^−/−^ mice failed to prevent spontaneous encephalomyelitis *in vivo*^52^.

Proximity imposed by Treg and Tconv peptide recognition on the same (F1) APC, renders suppression more complete (Fig. 4d). This mechanism may be partially active in cultures using two separate APCs due to the high density of Tregs. However, *in vivo* it seems less likely that Tregs and Tconvs responding to antigen on separate APCs will achieve sufficient proximity to induce suppression. In the lymph node there is a large excess of bystander CD4 and CD8 T cells, which decrease the proximity of Tregs and self-reactive Tconvs. In this light, one way to achieve Treg-Tconv proximity *in vivo* is for the Treg and Tconv to be stimulated by the same APC. Recent data using a two-photon microscopy for LN-live imaging showed that Tregs migrate at high velocity and engage both Tconv and DC in brief but frequent contacts. The authors conclude that, under non-inflammatory conditions, a LN-resident DC is in contact with at least one Treg for 36–47% of the time^53^.

Even though IL-2 scavenging is an attractive mechanism for suppression, our study does not exclude a role for suppressive mechanisms requiring cell-to-cell contact. It is well possible that Tregs, physically interact with Tconvs and mediate suppression, for example, through gap-junction formation, shunting high levels of inhibitory cyclic adenosine monophosphate (cAMP) from Tregs to Tconvs^36^. In addition, a requirement for Treg-Tconv proximity is also compatible with the Treg secreting suppressive mediators, which are more effective (i.e. at sufficiently high concentrations) at short distance from the Treg. Finally its worth pointing out that Treg-Tconv proximity is not an absolute requirement for suppression, since Tregs display some activity under conditions of reduced proximity (Fig. 4d,e).

Additional work is required to fully characterize the multiple mechanisms of Treg-mediated suppression, but monoclonal Tregs may be a useful experimental tool for future studies.

## Material and Methods

### Mice

Foxp3 Tg mice on a C57BL/6 background were described previously^43^ and kindly provided by S. F. Ziegler (Seattle, USA). B3K506 TCR Tg Rag^−/−^ mice on a C57BL/6 background^44^ were kindly provided by P. Marrack and J. Kappler (Denver, USA). B3K506 TCR Tg Foxp3 Tg mice were generated in our lab by crossing B3K506 TCR Tg Rag^−/−^ to Foxp3 Tg animals. Foxp3^EGFP^ reporter mice on a C57BL/6 background^54^ were kindly provided by B. Malissen. ABM (anti-bm12) TCR Tg mice on a C57BL/6 background were described previously^55^. IL-2^EGFP^ Reporter mice on a C57BL/6 background^56^ were kindly provided by A. Freitas (Paris, France). OT-II TCR Tg Rag^−/−^ IL-2^EGFP^ reporter mice were generated in our lab by crossing OT-II TCR Tg Rag^−/−^ to IL-2^EGFP^ reporter animals. C57BL/6 OT-II TCR Tg mice, C57BL/6 Ly5.1, C57BL/6 Ly5.2, B6.H2-I-Abm12 (bm12) and BALB/c mice were purchased from The Jackson Laboratory (Bar Harbor, Maine, USA). C57BL/6.CD3ε-deficient mice and BALB/c Ly5.1 HA TCR Tg mice^57^ were kindly provided by A. Rolink (Basel, Switzerland). All adult mice were 6–12 weeks old and bred in our colony (University Hospital Basel); all animal experiments were carried out using procedures approved by the Cantonal Veterinary Office in Baselstadt and in accordance with the Cantonal and Federal laws of Switzerland. The Cantonal Veterinary Office of Basel-Stadt, Switzerland, approved the animal protocols.

### Media, Antibodies and reagents

All cells were grown in RPMI 1640 (Gibco /Lifetechnologies) supplemented with 10% heat-inactivated FCS. Biotin-conjugated anti-CD3 (145-2C11), Biotin-conjugated anti-CD4 (RM4-5), Biotin-conjugated anti-CD8 (53-6.7), PE-conjugated anti-CD45.2 (104), Alexa700-conjugated anti-IFNγ (XMG1.2), PerCP-conjugated anti-CD45.1 (A20), FITC-conjugated anti-CD69 (H1.2F3), FITC-conjugated anti-CD44 (IM7), APC-conjugated anti-CD19 (1D3), PE-conjugated anti-CD62L (Mel-14), PE-conjugated anti-CD5 (53-7.3), PerCP-conjugated anti-CD3 (145-2C11), Alexa700-conjugated anti-CD4 (RM4-5), APC-conjugated anti-CD11a (M17/4), PE-conjugated anti-TCRValpha 2 (B20.1), PE-conjugated anti-TCRVbeta 5 (MR9-4) and APC-conjugated anti-TCRVbeta 8 (MR5-2) were purchased from BD Pharmigen (www.bdbioscience.com). PerCP-conjugated anti-NRP-1 was purchased from R&D Systems Inc. (www.RnDSystems.com). PE-Cy7-conjugated anti-GITR (DTA-1), PE-conjugated anti-TBET (4B10), PE-Cy7-conjugated anti-Foxp3 (FJK-16a) and APC-conjugated anti-Helios (22F6) was purchased from eBioscience (www.eBioscience.com). Pacific blue-conjugated anti-CD4 (RM4-4), Alexa700-conjugated anti-CD86 (GL-1), APC-conjugated anti-LAP (TW7-16B4), Alexa700-conjugated anti-CD25 (PC61) and APC-conjugated anti-CTLA-4 (UC10-4B9) were purchased from BioLegend (San Diego, CA, USA). EasySep Mouse CD4 + T cell Isolation Kit and EasySep Mouse B cell Isolation Kit, were purchased from Stemcell Technologies (www.stemcell.com). Recombinant Mouse IL-2 was purchased from BioLegend (San Diego, CA, USA). Cell Proliferation dye eFluor 670 was purchased from eBioscience (www.eBioscience.com). BD Cytofix/Cytoperm Plus Fixation/Permeabilization Kit with GolgiStop and BD Cytometric Bead Array Mouse Th1/Th2/Th17 CBA Kit was purchased from BD Pharmigen (www.bdbioscience.com). Anti–IL-2 (JES6-1) and anti-CD3 (145-2C11) were produced in our lab. Protein G Sepharose 4 Fast Flow, was purchased from (www.gelifescience.com). Peptides 3K (FEA QKA KAN KAV), P8G (FEA QKA KAN GAV), P2A (FEA AKA KAN KAV), and OVA (323-339) (ISQ AVH AAH AEI NEA GR), were purchased from AnaSpec (Fremont, CA, USA). HA-Peptide (YPY DVP DYA) was kindly provided by L. Klein (LMU, Germany). Dynabeads Biotin Binder, Cell trace CFSE and LIVE/DEAD fixable Near-IR were purchased from Invitrogen (Eugene, Oregon, USA). PMA and Ionomycin were purchased from Sigma-Aldrich.

### IL-2 complex treatment

B3K506 Treg mice were injected i.p. with 7.5 μg anti-IL-2 antibody (JES6-1) coupled to 2.5 μg mouse rIL-2 in 200 μl PBS on three subsequent days (see Supplementary Fig. S1). Complex formation was achieved by incubating IL-2 and JES6-1 in PBS for 30 min at 37 °C.

### Preparation and sorting of lymphocytes

LNs (axillary, inguinal, superficial cervical, mandibular, and mesenteric) were harvested from 6–12 week old mice. For single cell preparation, they were passed through a mesh into RPMI/10% FCS. B3K506 TCR Tg Foxp3 Tg cell suspensions were then incubated with Pacific Blue conjugated anti-CD4 and PE-Cy7 conjugated anti-GITR antibodies for 10 min at 4 °C, washed, and then sorted for CD4^+^ GITR^+^ cells on a BD INFLUX Cell Sorter (purity ≥96%, see Supplementary Fig. S1) into RPMI/10%FCS. B6 Foxp3 EGFP cell suspensions were sorted for GFP^+^ cells on a BD INFLUX Cell Sorter (purity ≥98%) into RPMI/10%FCS. Spleens were harvested from 6–12 week old mice. For single cell preparation they were passed through a mesh into erythrocyte lysis buffer and incubated for 1 min and subsequently washed in RPMI/10% FCS. T cell depletion was then preformed with the EasySep Mouse B cell Isolation Kit from Stemcell technologies (according to manufacture’s protocol) or with Dynabeads Biotin Binder Kit (according to manufacture’s protocol, Invitrogen) after incubating the splenocyte suspension for 10 min with biotin-conjugated anti-CD4, biotin-conjugated anti-CD8, biotin-conjugated anti-CD3 antibodies.

### *In vitro* suppression culture

10e5 monoclonal B3K506 Tregs, 10e5 polyclonal B6 FoxP3-EGFP Tregs or 10e5 monoclonal B3K506 Tconvs (control) were cultured in 96-well plates (0.2 ml) along with 2.5 × 10e4 OT-II Tconvs, 10e5 T cell–depleted splenocytes as a source of APCs and 0.1 μg/ml anti-CD3 or 3K, P8G or P2A peptides (at various concentrations, see figures). To experiment in Suppl. Fig. 2f, 50 ng/ml of recombinant Il-2 was added. T cell depleted splenocytes were preloaded with 10e-7M OVA (323-339) peptide for 4.5 h at 37 °C and washed 3x. OT-II Tconv were labeled with 5 μM CFSE or 5 μM eFlour 670 according to manufacture’s protocols. Flow cytometric analysis of suppressive cultures was preformed after 24, 48 and 72 h.

### Staining and Flow cytometry

Surface staining was preformed in PBS/3% FCS at 4 °C for 10 min with various antibodies. For intracellular staining, cells were fixed and permeabilized (according to manufacture’s protocol) using the Cytofix/Cytoperm Plus Fixation/Permeabilization Kit from BD. Intracellular Foxp3 staining was preformed at 4 °C for 1 h. For intracellular cytokine staining cultured cells were re-stimulated with 100 ng/ml PMA, 1.5 μM Ionomycin and 1.5 μl/ml Monensin (BD Pharmigen) and incubated for 5 h at 37 °C. Flow cytometry was preformed with a FACSCanto II from BD Pharmigen (www.bdbioscience.com).

### *In vivo* graft transplantation

Tail skin from B6.bm12 Rag^−/−^ mice was isolated and transplanted onto the back of B6 Rag^−/−^ mice and allowed to heal in for 14 days. The following day mice were injected with 2 × 10e4 ABM (I-Abm12 specific TCR Tg) Tconvs along with 2 × 10e5 Foxp3^EGFP^ polyclonal Tregs or 2 × 10e5 B3 monoclonal B3K506 Tregs. One group of mice received 30 μg 3K peptide/200 μl PBS injected i.p. every second day until day 15 and a second group received no peptide. A control group was injected with 2 × 10e4 ABM Tconvs alone. Graft rejection was checked every 2nd day.

### Cytokine assay

Culture supernatants were stored at −80 °C and thawed. The BD Cytometric bead array (CBA) system using antibody-coated capture beads was used to quantitate various cytokines in the culture supernatants (see manufacture’s protocol). Analysis was preformed with Excel software (version 14.4.3) calculating unknown sample concentrations from a standard curve.

### Determination of CD69 up regulation on B3K506 Tconv cells using various peptides

10e5 T cell depleted splenic B cells (as a source of APCs) were isolated and loaded with varying amounts of peptide for 4.5 h at 37 °C before addition of 2.5 × 10e4 B3K506 Tconvs. Cells were cultured in 96-well plates (0.2 ml) with RPMI/10% FCS for 24 h at 37 °C. T cells were then surface stained (described above) for CD3, CD4 and CD69 and flow cytometry was preformed. EC50 values for CD69 upregulation were calculated using a nonlinear regression curve (log [agonist] vs. response; three parameters) using Prism, version 6.0b.

### Suppression experiments using same or separate APCs

For “same APC” cultures, 10e5 B3K506 Tregs (I-A^b^ restricted), 2.5 × 10e4 HA Tg Tconvs (I-E^d^ restricted) and 10e5 splenic B cells from BALB/c x B6 F_1_ mice (co-expressing I-E^d^ and I-A^b^) were cultured in a 96-well plate in 0.2 ml RPMI/10%FCS at 37 °C. For “separate APC” cultures, 10e5 B3K506 Tregs (I-A^b^ restricted), 2.5 × 10e4 HA Tg Tconvs (I-E^d^ restricted), 10e5 B6 splenic B cells (I-A^b^) and 10e5 BALB/c splenic B cells (I-E^d^) were cultured in 96-well plates in RPMI/10%FCS at 37 °C. 3K peptide (10e-7M) and HA peptide (10e-5M) was added to same and separate APC cultures. Cultures were analysed using flow cytometry after 72 h.

### Statistics

Cell proliferation Index (PI) was calculated using FlowJo software (version 9.7.7). % Suppression was calculated using the formula (100-(% proliferated Tconv from suppressed culture/% proliferated Tconv)*100). Curve fitting and statistical analysis was performed using Prism version 6.0b and Excel version 14.4.3.

## Additional Information

**How to cite this article**: Gubser, C. *et al.* Monoclonal regulatory T cells provide insights into T cell suppression. *Sci. Rep.* **6**, 25758; doi: 10.1038/srep25758 (2016).

## Supplementary Material

Supplementary Information
