# Supplementary material for: Monoclonal regulatory T cells provide insights into T cell suppression
Source: Sci Rep. 2016 May 23;6:25758. doi: 10.1038/srep25758 (PMC4876466; doi:10.1038/srep25758)

Monoclonal regulatory T cells provide insights into T cell suppression

Céline Gubser[1], Mathias Schmaler[2], Simona W. Rossi[3], and Ed Palmer[1*]

[1] Departments of Biomedicine and Nephrology, University Hospital Basel and University of Basel, 4031 Basel, Switzerland.

[2] Laboratory of Experimental Immunology, Department of Biomedicine, University Hospital Basel and University of Basel, 4031 Basel, Switzerland

[3] Laboratory of Regulatory Immunology Department of Biomedicine, University Hospital Basel and University of Basel, 4031 Basel, Switzerland.

Correspondence: ed.palmer@unibas.ch

Supplementary figure 1

a) Representative flow cytometry plots show CD69 expression on CFSE labelled OT-II Tconvs from suppressive (-----) and non-suppressive (-----) cultures at a $1_{Tconv}/4_{Treg}$ ratio at 72h. Numbers in the plots depict % of cells in each quadrant, n=3- 5.

b) Representative flow cytometry plots show CD25 expression on CFSE labelled OT-II Tconvs from suppressive (-----) and non-suppressive (-----) cultures at a $1_{Tconv}/4_{Treg}$ ratio at 72h, n=3-5. Numbers in the plots represent % of cells in each quadrant, n=3-5.

c) Representative histograms show CD44, CD62L, CD69, Tbet, CD5 and CD25 expression on OT-II Tconvs in suppressive (with 3K peptide, -----) vs. non-suppressive (without 3K peptide, -----) co-cultures at a $1_{Tconv}/4_{Treg}$ ratio at 72h.

d) Inguinal lymph nodes from PBS injected (top) or IL-2 complex (IL-2C) injected (bottom) B3K506 Treg mice.

e) Bar graph shows mean number +/- SEM of LN cells isolated from PBS injected (■) or IL-2 complex (IL-2C) injected (□) B3K506 Treg mice, n=3. f) Bar graph and representative flow cytometry plots show mean % +/- SEM of Foxp3+ LN T cells isolated from mice described in D, n=3. Numbers in the plots depict % of cells in quadrant.

g) Representative flow cytometry plots of B3K506 Tregs pre- and post- cell sorting, which was carried out based on CD4 and GITR expression. Numbers in the plots depict (%) of cells in gate/quadrant.

Supplementary figure 2

a) Graph shows mean % of CD69+ B3K506 Tconvs +/-SEM in response to various peptide antigens used at various concentrations. Cells were cultured for 24 hours, n=3.

b) Bar graph shows mean OT-II proliferation index +/-SD from suppressive co-cultures at a $1_{Tconv}/4_{Treg}$ ratio at 72h, where B3K506 Tregs were stimulated with various altered peptide ligands at their EC50 concentration (see A), n=8.

c) Representative histogram shows CD25 expression on unstimulated B3K506 Tregs with and without addition of exogenous IL-2 (1ng/ml) after 24 hours.

d) Representative flow cytometry plots show Foxp3 and LAP expression on OT-II Tconvs and B3K506 Tregs from suppressive cultures at a $1_{Tconv}/4_{Treg}$ ratio at 72 hours. Numbers in the plots represent % of cells in each gate.

e) Representative histogram shows CFSE-labelled I-E^d restricted HA Tconvs in culture with I-A^b expressing B6 splenic B cells after 72 hours.

f) In vitro anti-CD3ε suppression assays with (----) and without (- - - -) 50ng/ml recombinant IL-2. Representative proliferation of CFSE labelled polyclonal $CD4^+$ Tconvs co-cultured with polyclonal B6 $Foxp3^{EGFP}$ Tregs (----), monoclonal B3K506 Tregs (----) or B3K506 Tconv (----) at a $1_{Tconv}/4_{Treg}$ ratio, at 72h.

Supplementary figure 1

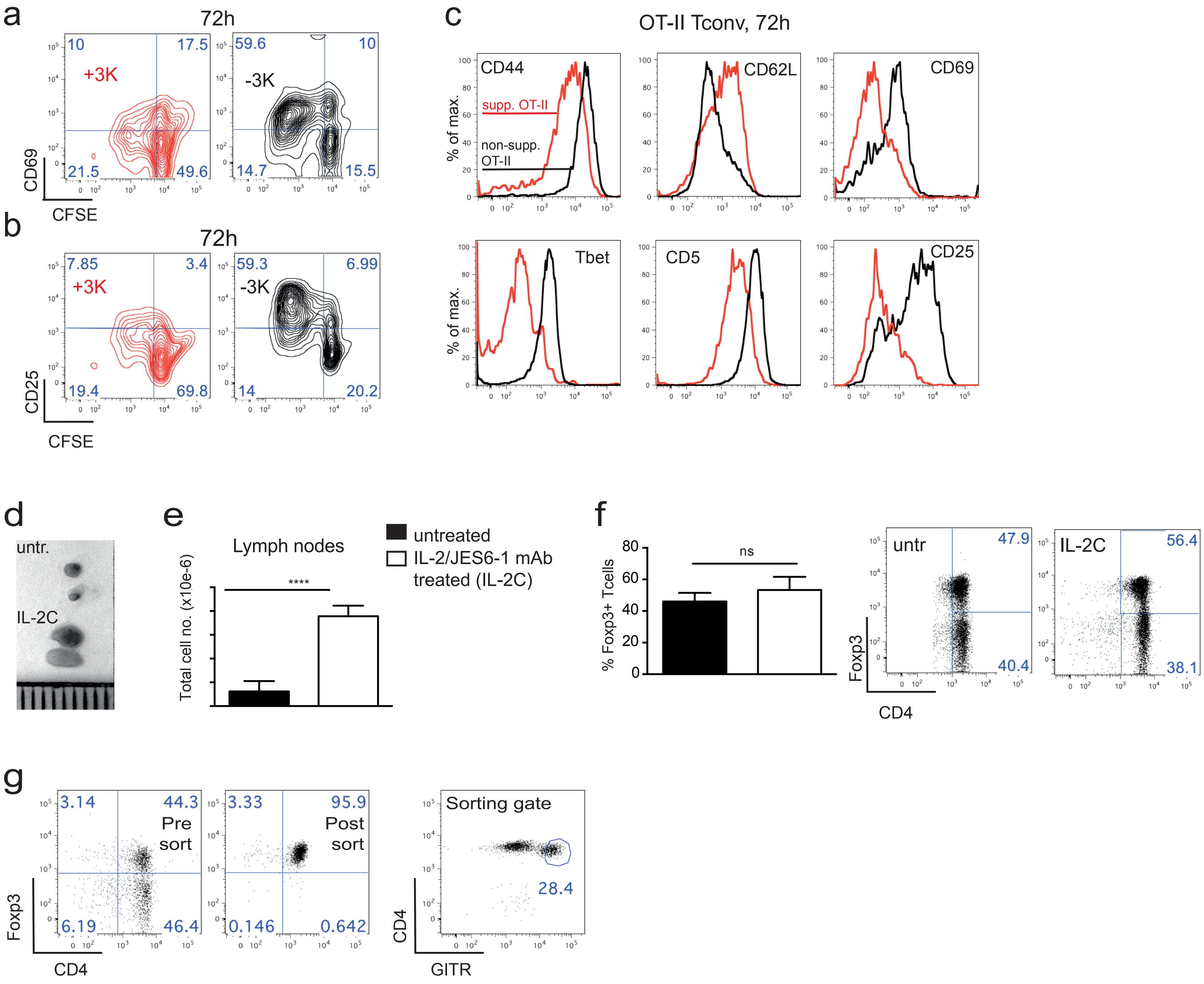

Supplementary figure 2

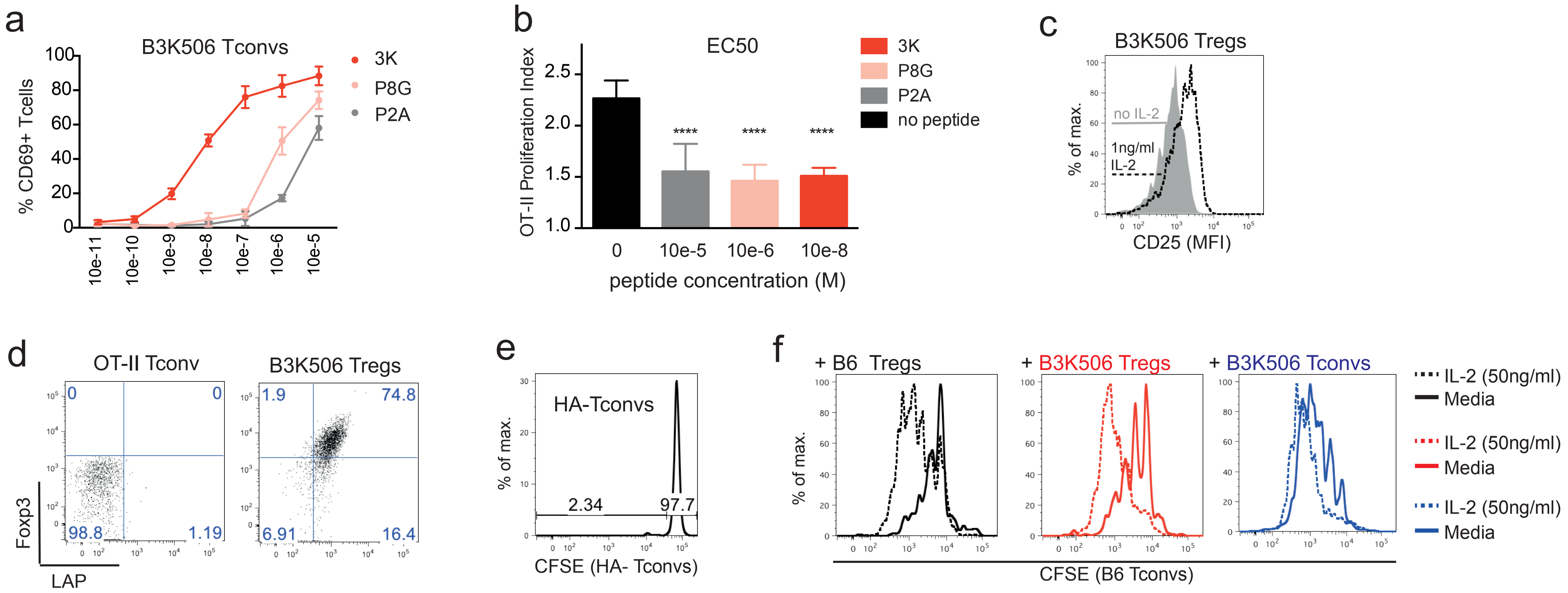

Supplement: Supplementary Information [file srep25758-s1.pdf]
